# Integrated transcriptomic and metabolomic analysis reveals the molecular profiles of dynamic variation in *Lilium brownii* var. *viridulum* suffering from bulb rot

**DOI:** 10.3389/fgene.2024.1432997

**Published:** 2024-08-14

**Authors:** Nana Chang, Lingling Zheng, Yang Xu, Chu Wang, Hui Li, Ye Wang

**Affiliations:** ^1^ Jiangxi Province Key Laboratory of Sustainable Utilization of Traditional Chinese Medicine Resources, Institute of Traditional Chinese Medicine Health Industry, China Academy of Chinese Medical Sciences, Nanchang, China; ^2^ Jiangxi Health Industry Institute of Traditional Chinese Medicine, Nanchang, China; ^3^ Institute of Chinese Materia Medica, China Academy of Chinese Medicinal Sciences, Beijing, China

**Keywords:** Lilium brownii var. viridulum, bulb rot, transcriptomic, metabolomic, combined analysis

## Abstract

*Lilium brownii* var. *viridulum*, known as Longya lily, is a well-known medicinal and edible plant in China. Bulb rot is a common disease in Longya lily cultivation that severely affects the yield and quality of lilies. According field investigations, we found that different Longya lily plants in the same field had different degrees of resistance to root rot. To find the reasons leading to the difference, we performed metabolomic and transcriptomic analyses of Longya lily with different degrees of disease. The transcriptomic analyses showed that the number of differentially expressed genes increased in early and mid-stage infections (LYBH2 and LYBH3), while decreased in late-stage infection (LYBH4). A total of 2309 DEGs showed the same expression trend in diseased bulb compared healthy bulb (LYBH1). The transcription factors (TFs) analysis of DEGs showed that several common TFs, like WRKY, bHLH, AP2/ERF-ERF and MYB, were significantly activated in bulbs after decay. The metabolomic analyses showed that there were 794 differentially accumulated metabolites, and metabolites with significant changes in relative content largely were phenolic acids, followed by flavonoids and amino acids and derivatives. The combined analysis of transcriptome and metabolome indicated that phenylpropanoid biosynthesis pathway was crucial in Longya lily resistance to bulb rot. Therefore, we speculated that the different degree of resistance to bulb rot in Longya lily may be related to the transcript levels of gene and contents of metabolites in the phenylpropanoid biosynthesis pathway. Overall, these results elucidate the molecular responses of Longya lily to bulb rot and lay a theoretical foundation for breeding resistant varieties.

## 1 Introduction

*Lilium brownii* var. *viridulum* Baker, a perennial bulbous herb, is a mutant variety of *L. brownii* F. E. Brown ex Miellez within the *Lilium* genus of the Liliaceae family. Commonly known as the Longya lily, meaning dragon’s teeth in Chinese, this plant is characterized by bulbs made up of many thick scales, with wide bases and pointed, curved tips. Its bulbs contain phenols, saponins, sterols, and alkaloids, which known for their sedative, anti-inflammatory and antitussive properties (Yang et al., 2019). Nevertheless, with the expansion of cultivation area and the increase of continuous cropping years, the occurrence of Longya lilies disease has gradually increased, especially bulb rot. The bulb rot, is commonly caused by soilborne fungal pathogens such as *F. oxysporum* f. sp. *lilii* (*F. oxysporum*), *Fusarium solani*, *F. proliferatum*, and *curvularia pseudobrachyspora* that become an important factor limiting the large-scale planting of Longya lilies (Pan et al., 2003; Li et al., 2018; Ren et al., 2020; Zeng et al., 2020). During the early stage of bulb rot disease, the scales appear brown slightly concave spots, which gradually expand and necrosis. In the later stage, the bulb browns and decays, the scales rot and peel off from the bulb disk, the leaves wilt and eventually the whole plant dies (Jiao et al., 2021). According to the previous statistics, this disease is highly prevalent in Wanzai, Taihe and Gao’an in Jiangxi, with a loss rate of 20%–30% in production, and can reach more than 60% in severe cases, which causes huge economic losses. The disease is difficult to prevent and control due to it is affected by complex environmental factors, such as temperature for pathogen growth, moderate to high soil moisture content, soil compaction, poor drainage, and so on. To alleviate losses caused by bulb rot, chemical pesticides are used many times and in large quantities, thus making pathogenic bacteria resistant and polluting the environment (Liao et al., 2013; Peng et al., 2015). Therefore, understanding the molecular mechanism of disease resistance, and breeding resistant varieties are the most economical and effective measures to prevent bulb rot.

Plant responses to pathogen infection trigger significant changes in gene expression and metabolism (Lu et al., 2020; Shen et al., 2023; Zhang et al., 2023). Transcriptome analysis, facilitated by RNA-Seq technology, allows for gene expression profiling in pathogen-infected plants, particularly those lacking a reference genome (Sun et al., 2019; Zhang et al., 2021a). This approach serves as a valuable tool to investigate how plants respond to various stresses, both abiotic and biotic, shedding light on the molecular mechanisms underlying their ability to withstand such conditions (Sun et al., 2019; Lu et al., 2020; Zhang et al., 2021a; Shen et al., 2023; Zhang et al., 2023). Additionally, metabolomics, which focuses on small molecular components, the combined transcriptome and metabolome analysis is widely used to uncover molecular mechanisms of plant response to biological or abiotic stresses. For instance, in resistant eggplant varieties, the metabolisms of phytohormones, phenylpropanoids, and flavonoids were found to be differentially enriched, highlighting their crucial role in defense against bacterial wilt (Xiao et al., 2023). Similarly, resistant broccoli showed significant increases in three indolic glucosinolates (I3G, 4MI3G, and 1MI3G) following infection with *Alternaria brassicicola*, underscoring the importance of these compounds in defense mechanisms (Shen et al., 2023). Flavonoid metabolism was identified as a key player in conferring resistance to stem canker in *Zanthoxylum bungeanum* (Li et al., 2021). Previous research on bulb rot disease primarily focused on pathogen isolation and identification, and the pathogenic mechanism of the pathogen, and so on (Pan et al., 2003; Ai et al., 2017; Li et al., 2018; Ren et al., 2020; Zeng et al., 2020). However, the impact of bulb rot on Longya lily at the transcriptional and metabolic levels, as well as the underlying response mechanisms, remains unclear.

In this study, we collected Longya lily bulbs with different degrees of disease from the same field, and found the key genes and metabolites associated with disease response through the combined transcriptome and metabolome analysis. Furthermore, the potential resistance mechanisms was speculated through the function of key genes and metabolites. These identified genes and metabolites can not only serve as valuable references for early disease diagnosis of Longya lily bulbs, but also provide theoretical basis for breeding disease-resistant varieties.

## 2 Materials and methods

### 2.1 Sample collection of Longya lily bulbs

The Longya lily used in this study was harvested in 2023 from Baishui township, located in the northwest of Wanzai County, Jiangxi Province, China, a widely cultivated species in the region. This field has been naturally infected with bulb rot disease and has since spread to large areas. In present research, we selected underground bulbs of Longya lily as the experimental materials. Four disease levels were divided in the experiment: LYBH1 (healthy: no obvious brown spots), LYBH2 (mild: brown spots occupied about 1/4 of the entire bulb), LYBH3 (moderate: brown spots occupied about 1/2 of the entire bulb), LYBH4 (severe: approximately the entire bulb turned brown and rotted). The textural and sensory were evaluated immediately after harvest, thereby defining the degree of disease. The surface of each scale was washed with clean water and sterilized with 75% ethanol, and then rinsed three times with sterile water. All fresh scales were cut into small pieces and immediately frozen in liquid nitrogen. Three biological replicates were collected for each level. Each replicate contained mixed sampled bulbs from at least five sampled sites. All bulb samples were sealed and sent for transcriptomic and metabolomic analyses.

### 2.2 RNA extraction and library construction

Total RNA was extracted using the Spin Column Plant Total RNA Purifcation Kit (Sangon Biotech, Shanghai, China), following the manufacturer’s instructions. The RNA quality was assessed by analyzing integrity with 1% agarose gel electrophoresis and the Agilent 2100 bioanalyzer, and concentration by the Qubit 2.0 Fluorometer. For cDNA library construction, total RNA ≥1 µg was used as a template with the Illumina NEBNext^®^ UltraTM RNA Library Prep Kit (NEB, Ipswich, MA, United States). Library quality was evaluated on the Agilent Bioanalyzer 2100 (Agilent, Santa Clara, CA, United States). Once qualified, all cDNA libraries were sequenced on the Illumina Hiseq 2000 platform (San Diego, CA, United States).

### 2.3 Reads mapping and functional annotation

Raw reads underwent quality control to obtain clean data by removing adaptors, reads with N content >10%, and low-quality bases (Q ≤ 20) exceeding 50%. High-quality reads were further filtered based on sequencing error rate and GC content distribution, then mapped to the assembled transcriptome for gene expression quantification in the absence of reference genomes. Clean reads were assembled into a reference sequence using Trinity (version 2.13.2) (Friedman et al., 2011).

Gene expression levels were quantified as fragments per kilobase per transcript per million mapped reads (FPKM) after normalization. Gene functions were annotated using databases such as the Kyoto Encyclopedia of Genes and Genomes (KEGG), Gene Ontology (GO), and NCBI non-redundant protein sequences (Nr). Differentially expressed genes (DEGs) were identified between groups using DESeq2 software (version 1.22.2) with criteria of |Log_2_fold change (FC)| ≥ 1 and false discovery rate (FDR) < 0.05 (Love et al., 2014; Varet et al., 2016). Subsequently, DEGs were further analyzed through clustering using the R software package (pheatmap: version 1.0.12). Gene functions were annotated using databases such as Kyoto Encyclopedia of Genes and Genomes (KEGG), Gene Ontology (GO) and NCBI non-redundant protein sequences (Nr). PlnTFDB (Perez-Rodriguez et al., 2010) and PlantTFDB (Jin et al., 2014) database was integrated to predict plant transcription factors (TFs) using iTAK software (version 1.7a) (Zheng et al., 2016).

### 2.4 Metabolites extraction

The freeze-dried samples were crushed using a mixer mill (MM 400, Retsch Technology, Haan, Ger-many) with a zirconia bead for 1.5 min at 30 Hz. The 50 mg of lyophilized powder was dissolved in 1.2 mL of 70% methanol solution and vortexed for 30 s at 30 min intervals for a total of 6 times, then centrifuged (12,000 rpm for 3 min). The supernatant was aspirated, and the sample was filtered through a microporous membrane (0.2 µm pore size) and stored in the sample vial for UPLC-MS/MS analysis.

### 2.5 Quantitative analysis of metabolomics

Sample extracts were analyzed using ultra-performance liquid chromatography electrospray ionization tandem mass spectrometry (UPLC-ESI-MS/MS) system (UPLC, Shim-pack UFLC SHIMADZU CBM30A system; MS/MS, Applied Biosystems 6500 QTRAP). Chromatographic separation was conducted on an Agilent SB-C18 column (1.8 µm, 2.1 mm × 100 mm, Agilent, New York, NY, United States) at 40°C. The UPLC analysis utilized a solvent system of ultrapure water (0.1% formic acid) and acetonitrile (0.1% formic acid) with a gradient program of 95:5 v/v at 0.0 min, transitioning to 5:95 v/v from 0.0 to 9.0 min, maintaining 5:95 v/v from 9.0 to 10.0 min, and returning to 95:5 v/v from 10.0 to 11.1 min, continuing until 14.0 min. The flow rate was set at 0.35 mL min^−1^, temperature at 40°C, and injection volume at 2 µL. The effluent was directed to an ESI-triple quadrupole-linear ion trap (QTRAP)-MS for further analysis. The ESI source operated at a source temperature of 550°C, ion spray voltage of 5,500 V (positive mode)/- 4,500 V (negative mode), and gas pressures of 50, 60, and 25 psi for ion source gas I, gas II, and curtain gas, respectively. Mass spectrometry data processing was performed using Analyst software (Version 1.6.3, AB Sciex, Fram-ingham, MA, United States). Metabolite analysis involved qualitative assessment based on secondary spectral information from the self-built database MWDB (Metware database) and quantitative analysis using multiple reaction monitoring (MRM) modes of triple quadrupole mass spectrometry.

### 2.6 Statistical analysis

Multivariate statistical analysis, including principal component analysis (PCA) and orthogonal partial least squares discriminant analysis (OPLS-DA), was performed using the R software package. The OPLS-DA models were evaluated based on their R2X, R2Y and Q2 values (R2: interpretation rate of the model, Q2: predictability of the model). The PCA plots were used to analyze the variability between groups and within groups. The OPLS-DA was performed to maximize the metabolome differences between sample pairs. The relative importance of each metabolite to the OPLS-DA model was tested using the variable importance in projection (VIP) as a parameter. For the VIP (VIP >1) value from OPLS-DA model, combined with absolute Log_2_FC (|Log_2_FC| ≥ 1.0) were used as the thresholds for screening differentially accumulated metabolites (DAMs). The functions of DAMs were further annotated using the KEGG compound database.

## 3 Results

### 3.1 Morphology of Longya lily bulbs with different degrees of disease

**FIGURE 1:**
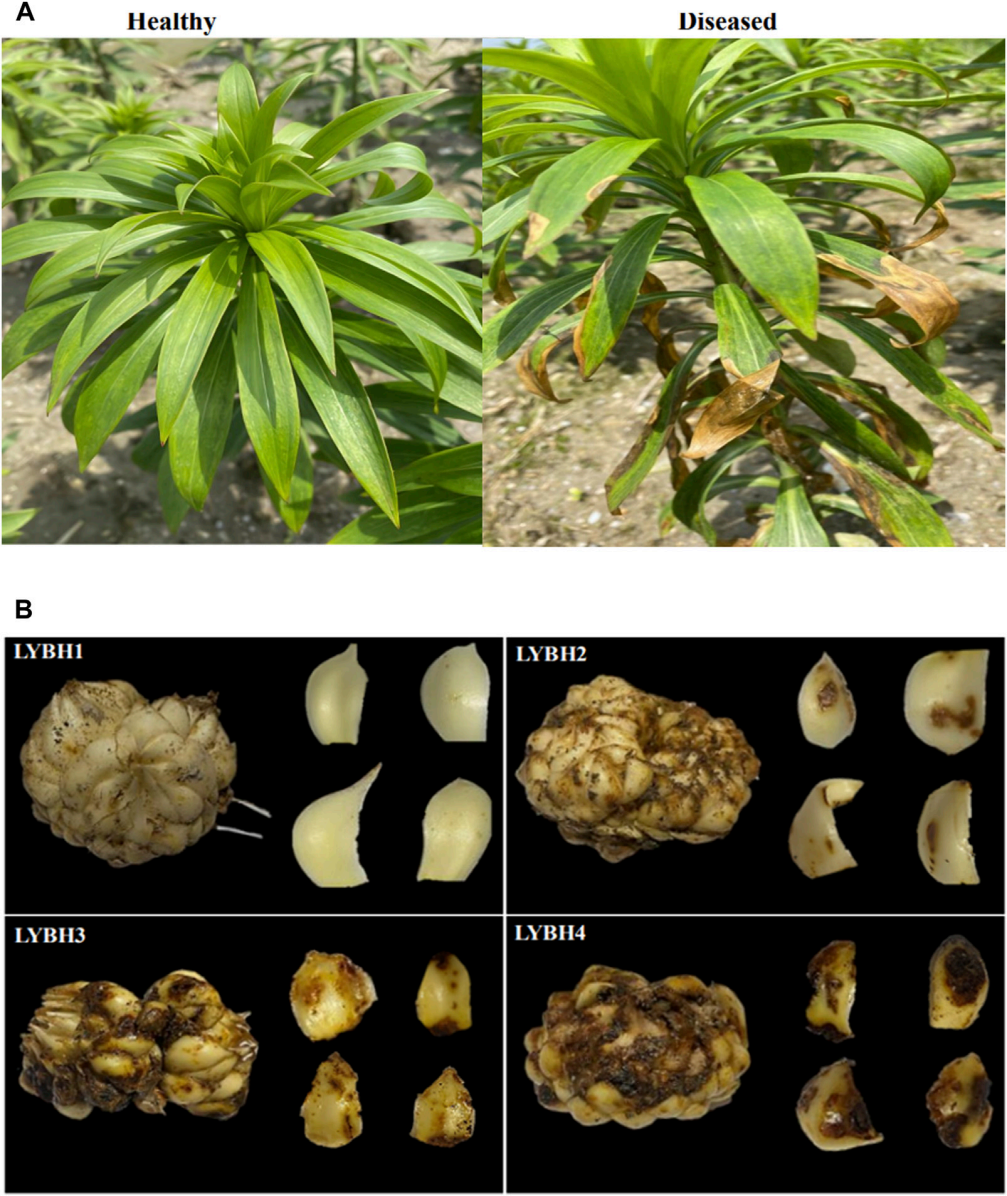
**(A)** Growth of healthy and diseased Longya lilies in the field. **(B)** Each disease level of Longya lily bulbs. LYBH1: Healthy; LYBH2: Mild; LYBH3: Moderate; LYBH4: Severe. Scale bars = 1.5 cm.

According to the field observations, we discovered that the Longya lily plants and bulbs with different disease levels had large phenotypic differences. The healthy plants were morphologically greener in color and exhibited normal growth, with no disease spots on the bulbs. In the early stages of the occurrence of the bulb rot disease, the edges of some leaves began to yellow, but the plant still grew normally, and a small number of brown spots appeared on the outer scales of the bulbs. With the development of diseases, a large number of leaves yellowing expanded and eventually covered the entire leaf, the surface of the bulbs had several sunken dark brown lesions and multiple lesions could merge with each other to appear water-stained. In the late stage of the disease, the leaves of the whole plant turned yellow or purple from bottom to up, and then wilted and dried up, and the base of the stem shrank, which was easy to break; the color of the lesions on the bulbs gradually darkened and deepened into the bulb tissue, creating a cavity, and then the bulbs completely deteriorated and formed mushy rot, eventually lead to death of the whole plants (Figures 1A, B). These performances were consistent with previous reports showing that lily infected bulb rot disease caused by *F. oxysporum*.

### 3.2 RNA sequencing and quality control

Based on quality assessment of the RNA extraction, the extracted concentrations were between 10 and 32 ng μL^−1^, and the integrity were from 6.3 to 8.6, indicating good RNA quality, which met the requirements for sequencing and library construction (Supplementary Table S1). A total of 99,711,225 raw reads were obtained by transcriptome sequencing, and 96,627,540 clean reads were derived after filtering the original data; altogether, 173.92 Gb of clean data was acquired. Each sample had over 12 Gb of clean data and the GC content was about 50.38%. The percentage of Q20 base was about 97.92%, and the Q30 base was about 94.09%, indicating the relatively high quality of transcriptome data (Supplementary Table S2). Ultimately, the sequence and expression information of 127,621 unigenes was obtained for further data analysis.

### 3.3 Differentially expressed genes analysis

**FIGURE 2:**
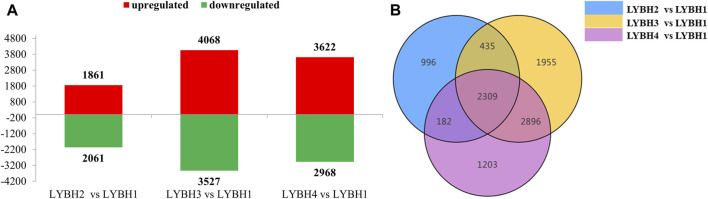
**(A)** Number of up/downregulated DEGs in the three comparison groups. **(B)** Venn diagram showing the DEGs numbers in each comparison group.

To investigate the gene expression changes induced by bulb rot disease in Longya lily, all samples were divided into three comparison groups: LYBH2 vs. LYBH1, LYBH3 vs. LYBH1 and LYBH4 vs. LYBH1. The differentially expressed genes (DEGs) for different comparisons were identified by DESeq based on FPKM values. A total of 9,976 DEGs were identified in three comparison groups. In LYBH2 vs. LYBH1, there were 3,922 DEGs (1,861 upregulated and 2,061 downregulated). Similarly, there were 7,595 DEGs (4,068 upregulated and 3,527 downregulated) were identified in LYBH3 vs. LYBH1, whereas the LYBH4 vs. LYBH1 group contained 6,590 DEGs (3,622 upregulated and 2968 downregulated) (Figure 2A). Venn diagram analysis showed that 2,309 (986 upregulated and 1,323 downregulated) DEGs were shared in three comparison groups, 996, 1,955 and 1,203 DEGs were unique to the LYBH2 vs. LYBH1, LYBH3 vs. LYBH1 and LYBH4 vs. LYBH1, respectively (Figure 2B). This result indicated that there was a significant change in gene expression levels between the healthy and infected Longya lily bulbs.

**FIGURE 3:**
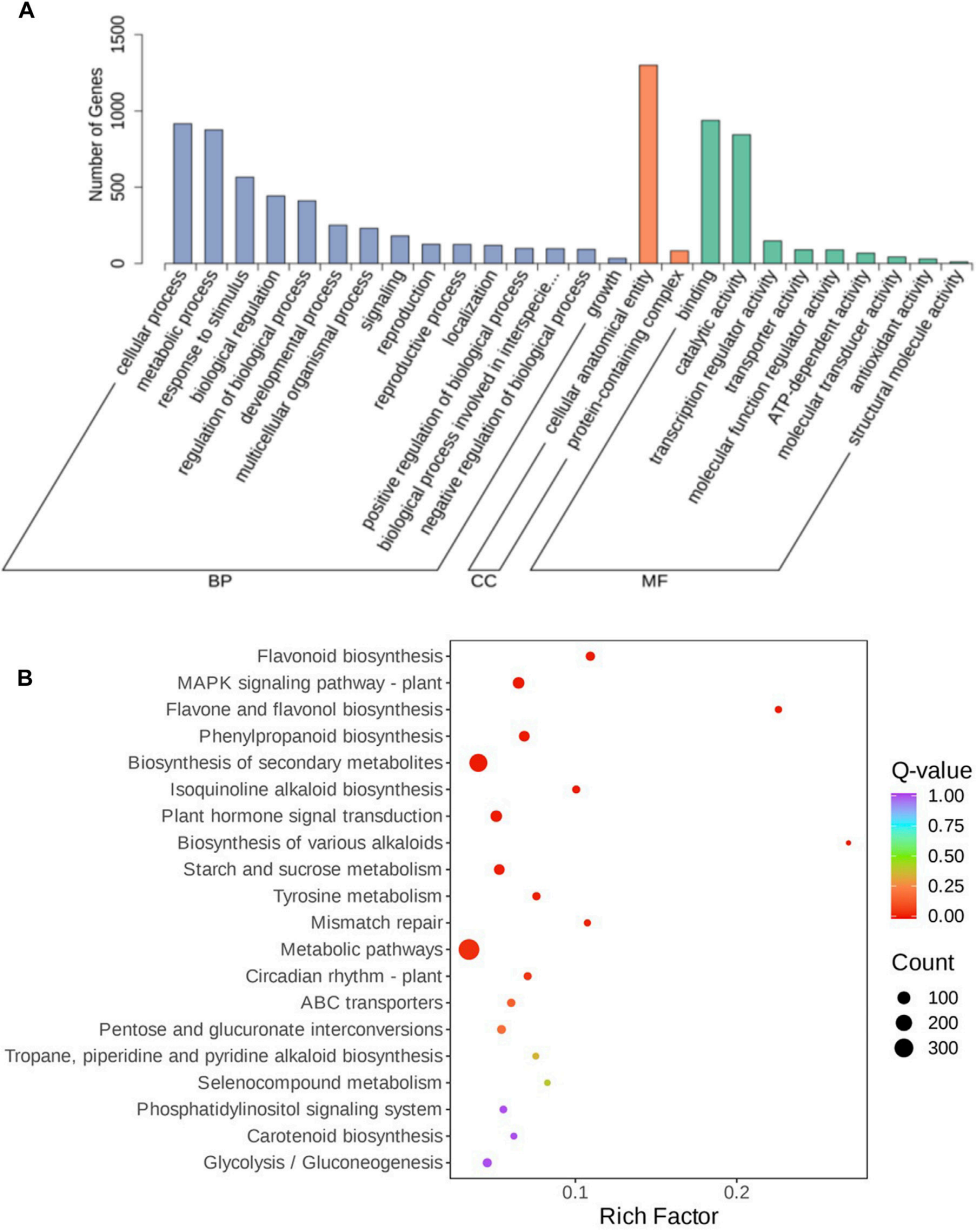
**(A)** GO enrichment analysis of DEGs from the three comparisons. **(B)** Top 20 enriched KEGG pathways of DEGs shared in the three comparison groups.

To evaluate the function of the shared DEGs between the three comparison groups, functional enrichment analyses using GO and KEGG database were carried out to identify possible biological processes or pathways involved in bulb rot disease infection response. All DEGs annotated in the GO database were divided into three categories: biological process (the top three GO terms were cellular process, metabolic process, and response to stimulus), molecular function (catalytic activity and binding, transcription regulator activity), and cellular component (cellular anatomical entity and protein-containing complex (Figure 3A). Furthermore, all DEGs were mapped to the KEGG pathways. According to the annotation results, we discovered that most DEGs were annotated to metabolic pathway, biosynthesis of secondary metabolites, MAPK signaling pathway-plant, plant hormone signal transduction, phenylpropanoid biosynthesis, starch and sucrose metabolism and plant-pathogen interaction pathways. The top 20 pathways with the lowest *p*-values showed that flavonoid biosynthesis, MAPK signaling pathway-plant, flavone and flavonol biosynthesis, phenylpropanoid biosynthesis, isoquinoline alkaloid biosynthesis, plant hormone signal transduction, biosynthesis of various alkaloids, starch and sucrose metabolism, tyrosine metabolism, mismatch repair and circadian rhythm-plant pathways were significantly enriched (Figure 3B), some of which have been previously reported to be related to plant disease resistance, like flavonoid biosynthesis, phenylpropanoid biosynthesis, plant hormone signal transduction, starch and sucrose metabolism, and so on. In addition, we performed KEGG enrichment analysis for DEGs unique to each group (Supplementary Table S3). In LYBH2 vs. LYBH1, a total of 113 pathways were enriched by specific DEGs, of which the glutathione metabolism and monoterpenoid biosynthesis pathways were significantly enriched. In LYBH3 vs. LYBH1, the number of pathways for specific DEGs enrichment was 124, and the phenylpropanoid biosynthesis, cyanoamino acid metabolism and plant hormone signal transduction pathways were significantly enriched. Correspondingly, a total of 109 pathways were enriched, and the protein processing in endoplasmic reticulum and carotenoid biosynthesis were significantly enriched among them. This indicated that the Longya lily in the different stage of infection defended against pathogen invasion by activating different metabolic pathways.

### 3.4 Transcription factors analysis of DEGs

Transcription factors (TFs) and transcriptional regulators (TRs) play an important regulatory role in plant growth, development and stress defence response. Therefore, we also analysed the TFs (TRs) in DEGs. In total, 52 TFs (TRs) from 270 DEGs were identified in the comparisons of LYBH2 vs. LYBH1; 59 TFs (TRs) from 524 DEGs were observed in the comparisons of LYBH3 vs. LYBH1, while 56 TFs (TRs) from 425 DEGs were found in the comparisons of LYBH4 vs. LYBH1. 46 common differentially expressed TFs (TRs) were identified in the three comparisons, such as WRKY, NAC, MYB, AP2/ERF-ERF, bHLH, bZIP, Tify, etc. These TFs (TRs) families have previously been reported to be involved in the regulation of various disease resistance pathways. Among them, the first two families with more TFs were bHLH and AP2/ERF-ERF family, which were all largely upregulated in the three comparisons. The C2C2-CO-like, HB-other, MADS-M-type and zf-HD TFs encoded by downregulated DEGs were unique in the LYBH2 vs. LYBH1 and LYBH3 vs. LYBH1 comparison groups. Some TFs (TRs) were only in the LYBH3 vs. LYBH1 and LYBH4 vs. LYBH1 com-parison groups, like AP2/ERF-RAV, BES1, C2C2-YABBY, E2F-DP, HB-KNOX and NF-YC. Also, CAMTA and TUB, were downregulated. Similarly, the RAF, LFY and DBP TFs (TRs) were downregulated only in LYBH3 compared to LYBH1, while the BBR-BPC and HMG, both upregulated and downregulated, were just discovered in the LYBH4 vs. LYBH1 comparison group. All data for the TFs can be found in Supplementary Table S4.

### 3.5 Overview of the metabolome

**FIGURE 4:**
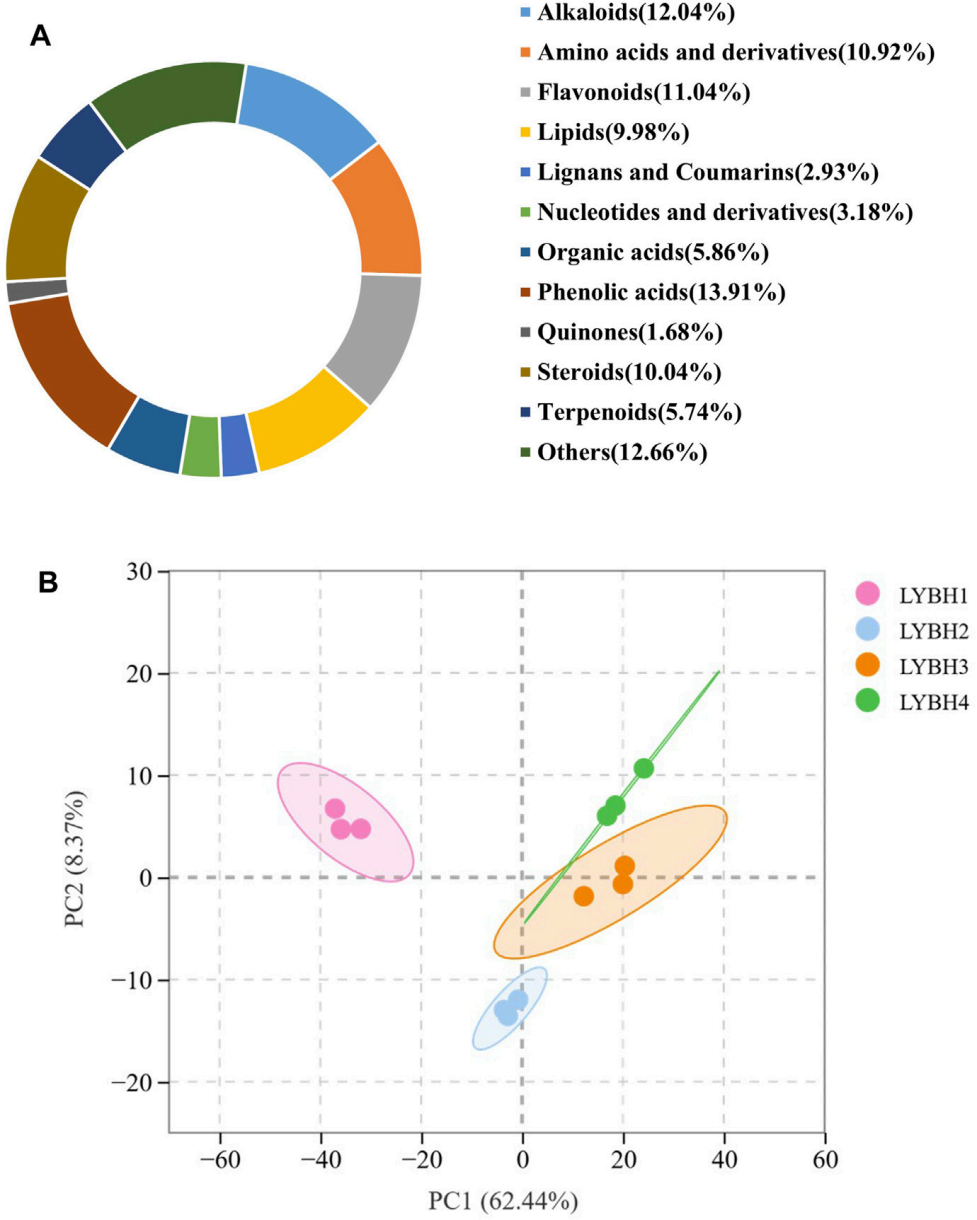
**(A)** Ring diagram of metabolites classification. **(B)** Principal component analysis (PCA) of all metabolites.

To gain insight into the metabolic changes and the possible defense mechanisms in the Longya lily bulbs after decay, widely targeted metabolomics by UPLC-MS/MS was performed to broadly evaluate metabolite profiles in healthy and diseased Longya lily bulbs. Based on UPLC-MS/MS analysis and a self-building database, a total of 1,603 metabolites were detected in all samples and divided into 11 categories. Among them, five categories account for more than 10% of the metabolites, including phenolic acids (13.91%), alkaloids (12.04%), flavonoid (11.04%), amino acids and derivatives (10.92%) and steroids (10.04%) (Figure. 4A). Principal component analysis (PCA) analysis of metabolomic profiles was conducted to provide information about distinctly separate groups among these samples of the four disease levels of Longya lily bulb and the three replicates of each disease level together. The two PC together explained 70.81% of the chemical variables. Based on PC1, 62.44% of the information could clearly distinguish LYBH1, LYBH2 and LYBH3, LYBH4 samples. The two-dimensional plot showed obvious variations in the metabolites from the bulbs of Longya lily after decay. However, the LYBH3 and LYBH4 samples had similar metabolic profiles perhaps due to the relatively similar severity of onset. Furthermore, three duplicate samples of each disease level grouped together, indicating high reproducibility among replicates (Figure 4B).

#### 3.5.1 Identification of differentially accumulated metabolites

**FIGURE 5:**
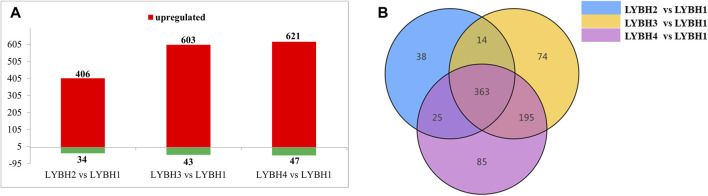
**(A)** Number of differentially accumulated metabolites detected in the three comparison groups. **(B)** Venn diagram showing DAMs numbers shared in three comparisons.

Based on VIP (VIP >1) and absolute Log_2_FC (|Log_2_FC | ≥ 1.0), there were 794 differentially accumulated metabolites (DAMs) were identified in three comparisons, of which 37 DAMs only accumulated in infected lily bulbs (LYBH2, LYBH3 and LYBH4), including 10 flavonoids, 5 lignans and coumarins, 4 steroids, 4 alkaloids, 3 phenolic acids, 2 terpenoids, 2 quinones, 1 organic acid, 1 nucleotides derivative and 5 other compounds. In LYBH2 vs. LYBH1, there were 440 DAMs (406 upregulated and 34 downregulated). Correspondingly, there were 646 DAMs (603 upregulated and 43 downregulated) in LYBH3 vs. LYBH1, and 668 DAMs (621 upregulated and 47 downregulated) in LYBH4 vs. LYBH1 (Figure 5A). And the most upregulated metabolites were phenolic acids, followed by lipids (Table 1). This indicated that phenolic compounds played key roles in in lily bulb rot resistance. In addition, a total of 363 DAMs were shared in three comparison groups, the number of DAMs that were unique to the comparison group of LYBH2 vs. LYBH1, LYBH3 vs. LYBH1 and LYBH4 vs. LYBH1 were 38, 74 and 85, respectively (Figure 5B).

**TABLE 1:** The detailed amount of difference metabolites in the three comparisons of lily.

Class	LYBH2 vs. LYBH1		LYBH3 vs. LYBH1		LYBH4 vs. LYBH1	
	Up	Down	Up	Down	Up	Down
Alkaloids	26	8	45	7	53	5
Flavonoids	40	10	49	14	53	21
Amino acids and derivatives	34	3	59	4	65	4
Organic acids	12	2	28	1	34	0
Phenolic acids	106	3	131	5	129	5
Terpenoids	35	0	46	1	44	1
Quinones	11	0	15	0	13	0
Lipids	50	1	74	1	76	0
Lignans and Coumarins	19	0	25	1	25	0
Nucleotides and derivatives	13	1	27	1	27	1
Steroids	15	5	23	4	18	6
Others	45	1	81	4	84	4
Sum	406	34	603	43	621	47

In this study, the top 10 DAMs with the largest absolute value of Log_2_FC were screened in three comparison groups, respectively (Supplementary Table S5). Among them, the upregulated phenolic acids (p-Coumaroylmalic acid and 1-O-p-Coumaroyl-β-D-glucose), amino acids derivatives (N-[malonyl] phenylalanine), and other substances (juglanoside D) were shared in the three comparison groups. Apart from this, the top 10 DAMs in LYBH2 vs. LYBH1 group included three upregulated phenolic acids (1-Feruloyl-3-p-coumaroyl-2′,6′-diacetylsucrose, vanilloloside and 6-O-p-Coumaroyl-β-D-glucose*), one upregulated flavonoid (kaempferol-3-O-rutinoside-7-O-rhamnoside), one upregulated quinone (7-hydroxy-4-methoxyphenanthrene-2-O-glucoside) and one upregulated alkaloid (N′,N″-Diferuloylspermidine); the top 10 DAMs in LYBH3 vs. LYBH1 had two upregulated amino acids derivatives (jasmonoyl-L-isoleucine and 2′-deoxyadenosine), one upregulated flavonoid (choerospondin), one upregulated lipid (linoleoyl ethanolamine) and one upregulated alkaloid (N′, N″-Diferuloylspermidine), and one downregulated flavonoid (quercetin-3-O-glucoside (isoquercitrin)); the top 10 DAMs in LYBH4 vs. LYBH1 included three upregulated flavonoids (galangin-7-O-glucoside, apigenin-5-O-glucoside and choerospondin), one upregulated phenolic acid (ethyl ferulate) and one upregulated lipid (linoleoyl ethanolamine), and one downregulated flavonoid (quercetin-3-O-galactoside (hyperin)). Most of these compounds metioned above belong to phenolic acids, followed by flavonoids and amino acids and derivatives, and a few belong to lipids and alkaloids.

#### 3.5.2 KEGG enrichment analysis of DAMs

**FIGURE 6:**
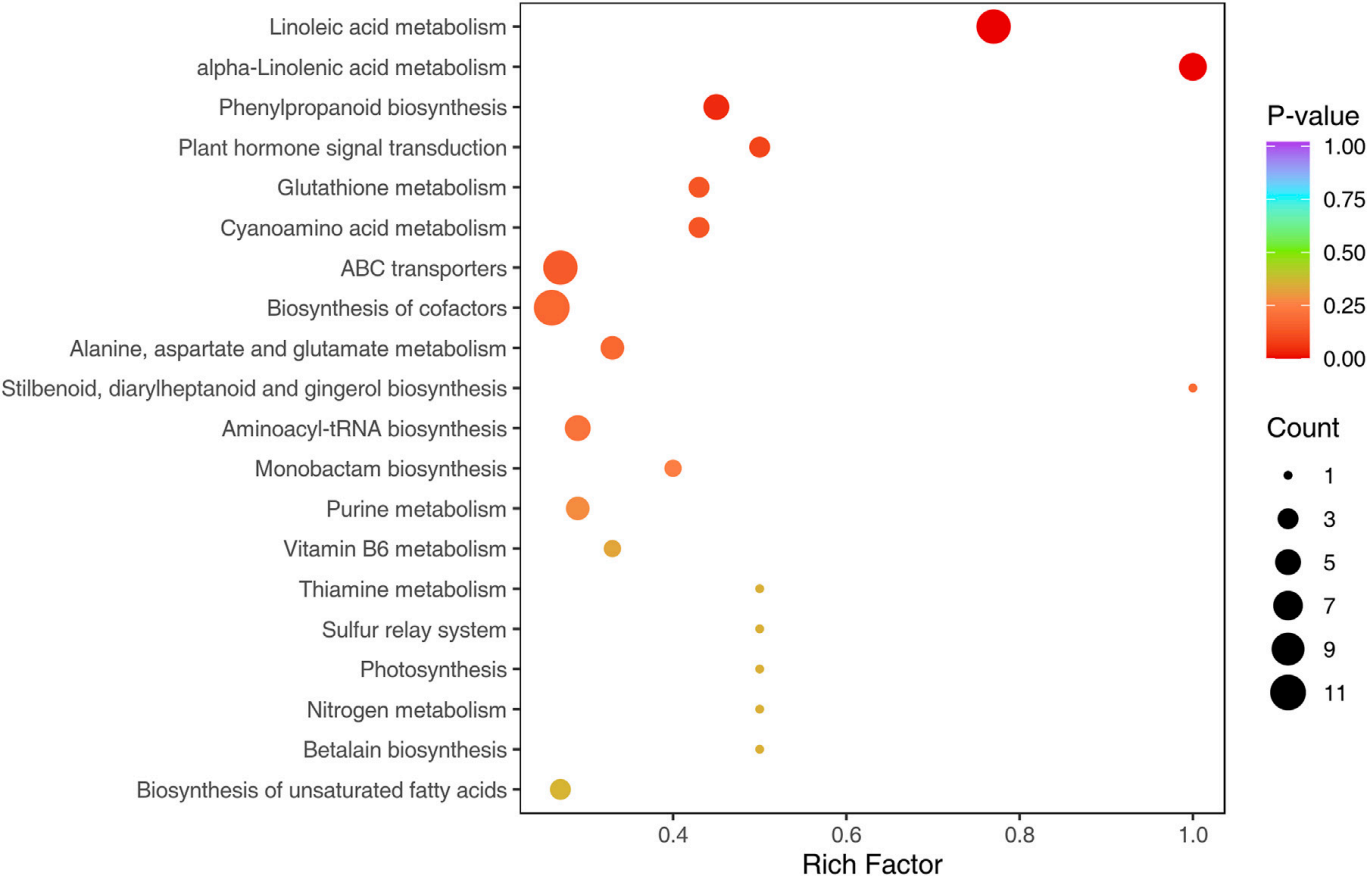
Top 20 enriched KEGG pathways of DAMs shared in the three comparison groups.

Based on KEGG annotations of the shared DAMs from the three comparisons, we discovered that a total of 63 pathways were annotated by all shared metabolites, the pathways with the highest number of metabolites were metabolic pathways, biosynthesis of secondary metabolites, biosynthesis of cofactors, linoleic acid metabolism, ABC transporters, biosynthesis of amino acids, alpha-linolenic acid metabolism, aminoacyl-tRNA biosynthesis, phenylpropanoid biosynthesis and purine metabolism. The KEGG functional enrichment analysis showed that linoleic acid metabolism, alpha-linolenic acid metabolism, phenylpropanoid biosynthesis, plant hormone signal transduction, glutathione metabolism, cyanoamino acid metabolism and ABC transporters were significantly enriched, of which linoleic acid metabolism, alpha-linolenic acid metabolism and phenylpropanoid biosynthesis were extremely significantly enriched (*p* < 0.01) (Figure 6). Thus, we speculate DEMs involved in these metabolic pathways were essential in the Longya lily response to bulb rot.

### 3.6 Integrated transcriptomics and metabolomics analysis

**FIGURE 7:**
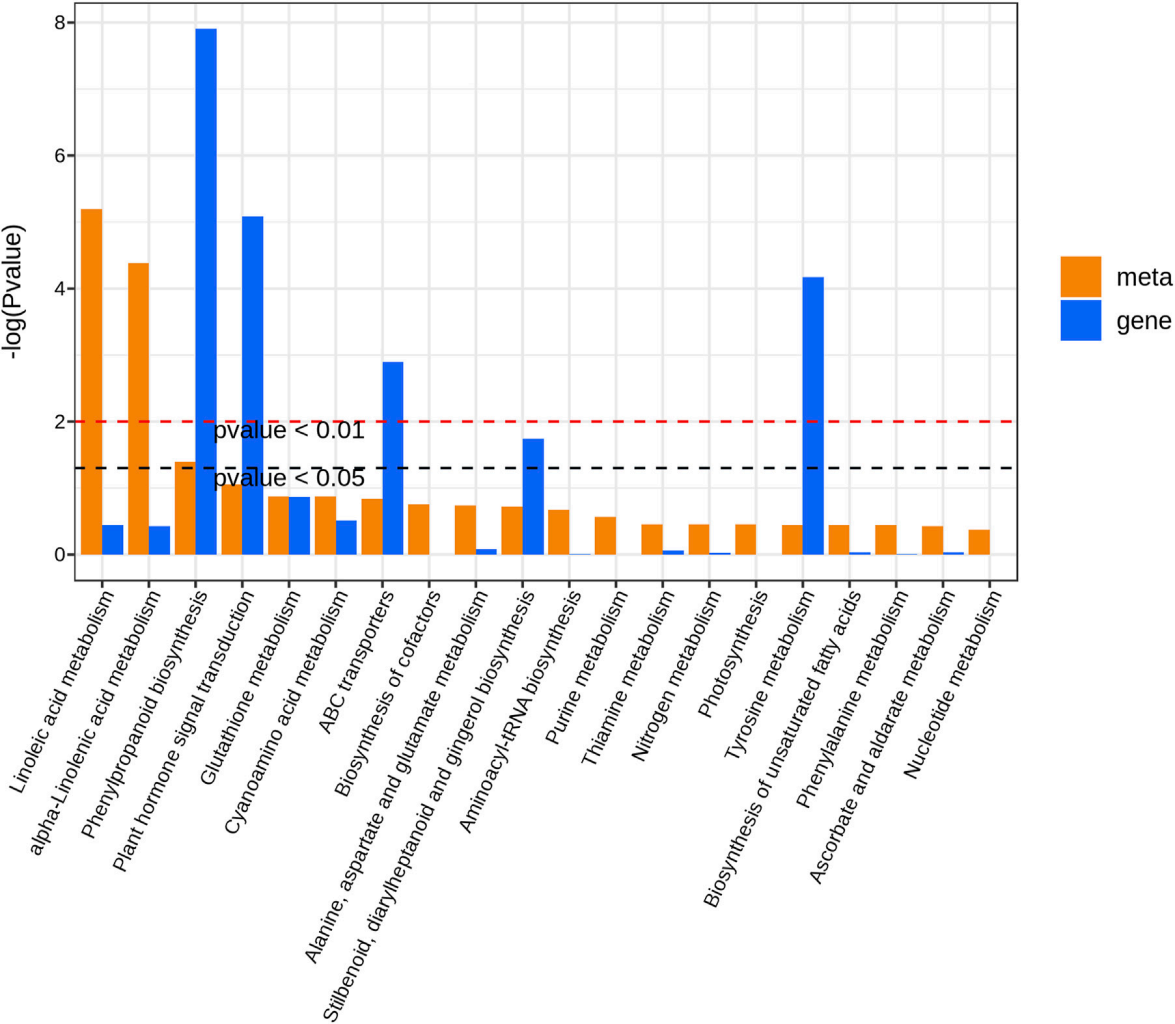
Co-enrichment analysis of DAMs and DEGs shared in the three comparison groups.

**FIGURE 8:**
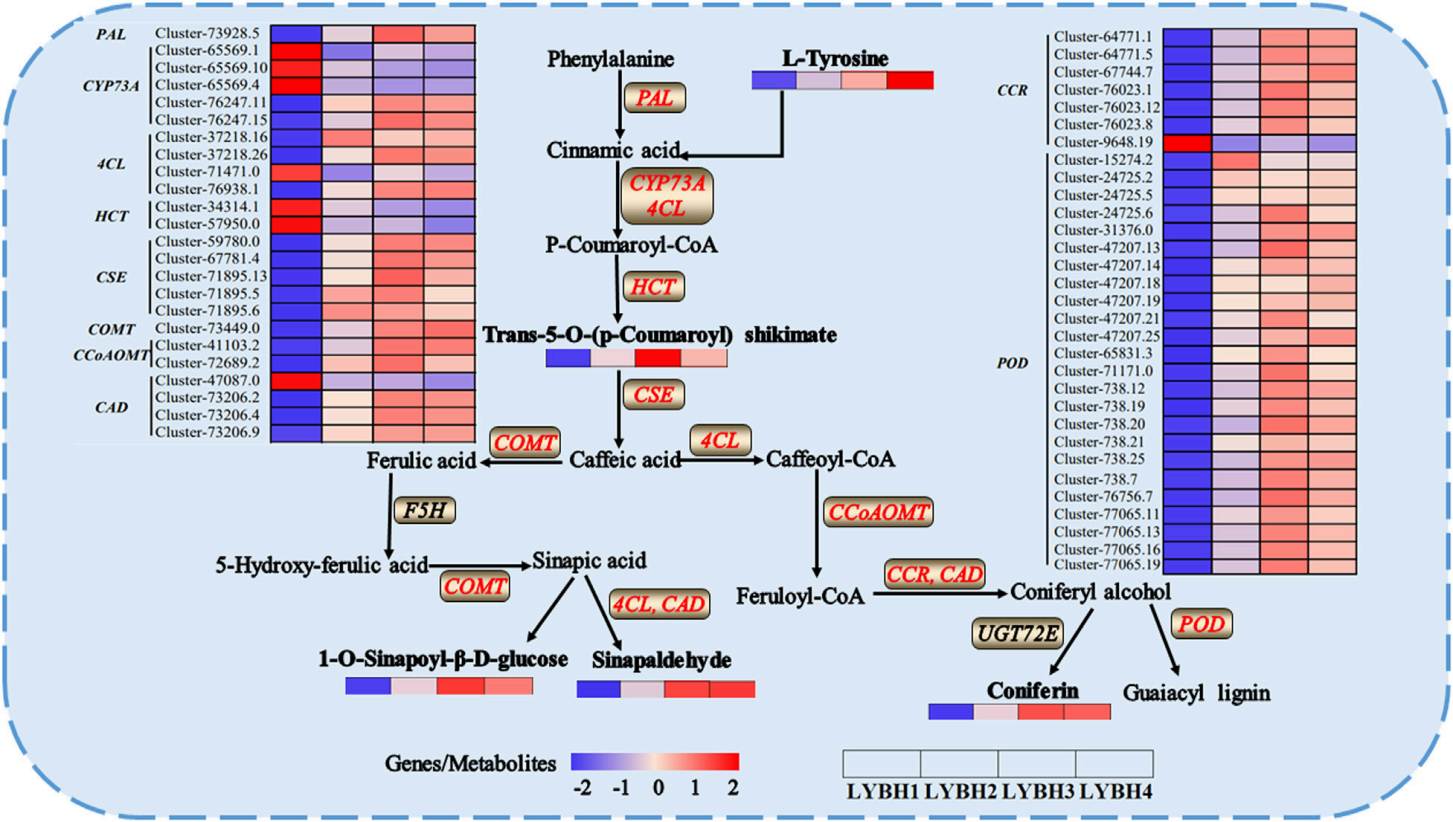
The schematic diagram of key genes and metabolites in the phenylpropanoid biosynthesis involved in Longya lily the response to bulb rot. The words in bold black indicated the DAMs in each sample. The words in red indicated the DEGs in each sample.

To further explore the relationship between DEGs and DAMs in Longya lily responsive to bulb rot disease, the co-joint KEGG pathway enrichment analysis of the shared 2309 DEGs and 363 DAMs in three comparison groups was conducted. Results showed that these DEGs and DAMs enriched to 115 and 62 pathways, respectively, whereas 51 pathways were shared by DEGs and DAMs. Then, we showed the 20 pathways most significantly enriched in terms of DEGs and DEMs of the pathways shared in three comparison groups with the lowest *p*-value (Figure 7). Among them, we found that linoleic acid metabolism, alpha-linolenic acid metabolism and phenylpropanoid biosynthesis were significantly enriched by DAMs, but the pathways significantly enriched by DEGs were phenylpropanoid biosynthesis, plant hormone signal transduction, ABC transporters, stilbenoid, diarylheptanoid and gingerol biosynthesis and tyrosine metabolism. Interestingly, only phenylpropanoid biosynthesis was significantly enriched in both the transcriptomic and metabolomic data. This result suggested that the phenylpropanoid biosynthesis pathway may be the main regulatory pathway involved in the Longya lily defenses against bulb rot disease. A network of the diagram was constructed to further analyze the relationship between DAMs and DEGs in the phenylpropanoid biosynthesis pathways (Figure 8). We found that there were five upregulated DAMs (L-tyrosine, trans-5-O-[p-coumaroyl] shikimate, 1-O-sinapoyl-β-D-glucose, sinapinaldehyde and coniferin), and 55DEGs involved in this pathway. Furthermore, we calculated Pearson correlations between DEGs and DAMs involved in phenylpropanoid biosynthesis pathway, retaining only strong (|r| (correlation coefficient) ≥ 0.8) and statistically significant (*p* < 0.05) associations (Supplementary Table S6). The results showed that the cinnamate 4-Hydroxylase (*CYP73A*) encoded by the cluster-65569.4 and cluster-65569.10 was negatively correlated with L-tyrosine, trans-5-O-(p-coumaroyl) shikimate, 1-O-sinapoyl-β-D-glucose, sinapinaldehyde and coniferin, while the *CYP73A* encoded by the cluster-76247.11 and cluster-76247.15 was positively correlated with trans-5-O-(p-coumaroyl) shikimate, 1-O-sinapoyl-β-D-glucose, sinapinaldehyde and coniferin. The shikimate O-hydroxycinnamoyl transferase (*HCT*) encoded by the cluster-34314.1 and cluster-57950.0 was negatively correlated with the sinapinaldehyde, trans-5-O-(p Coumaroyl) shikimate and 1-O-Sinapoyl-β-D-glucose, and the cinnamyl-alcohol dehydrogenase (*CAD*) encoded by the cluster-47087.0 was negatively correlated with the 1-O-Sinapoyl-β-D-glucose. The phenylalanine ammonia-lyase (*PAL*), 4-coumarate-CoA ligase (*4CL*), caffeoyl-CoA O-methyltransferase (*CCoAOMT*), cinnamoyl-CoA reductase (*CCR*), caffeic acid 3-O-methyltransferase (*COMT*), caffeoylshikimate esterase (*CSE*) and *peroxidase* (*POD*) encoded by the remaining DEGs were positively the five DAMs involved in the pathway. In addition, transcriptome analysis showed that the expression of some genes encoding the *CYP73A*, *4CL*, *HCT*, *CCR* and *CAD* decreased with the increase of disease severity, like the Cluster-65569.1, Cluster-65569.10, Cluster-65569.4, Cluster-71471.0, Cluster-34314.1, Cluster-57950.0, Cluster-47087.0 and Cluster-9648.19, while the expression of the genes encoding the *PAL*, *CCoAOMT*, *CSE*, *COMT* and *POD* increased.

## 4 Discussion

It has been reported that the bulb rot, mainly caused by *F. oxysporum*, is a common issue in lily cultivation and production, reducing harvest yield and quality, and subsequent death of severely infected plants (Li et al., 2018). This is a big challenge considering the multi-pathogenic cause of this disease and poor technologies for its management. Currently, this disease is controlled by a variety of methods, such as chemical pesticides, disease management in the field, plant breeding for resistant varieties/genetically modified plants, as well as biological control agents (Liao et al., 2013; Peng et al., 2015; Wu et al., 2015). With the rapid development of science and technology, the breeding of resistant varieties is considered the most economical and environmentally friendly long-term strategy for plant disease management. Therefore, it will become increasingly important to explore internal resistance genes to improve lily resistance. In this study, we performed transcriptome and metabolome analysis of Longya lily with different degrees of disease. Transcriptome analysis showed that there were 3922 DEGs in LYBH2 vs. LYBH1, 7595 DEGs in LYBH3 vs. LYBH1, 6590 DEGs in LYBH4 vs. LYBH1, and 2309 DEGs shared in three comparison groups, indicating complex and contrasting transcriptional changes in Longya lily induced by bulb rot disease. At the same time, we could see from the above data that the numbers of DEGs increased during early and mid-stage infections (LYBH2 and LYBH3), while decreased during late-stage infection (LYBH4). The reason for the results may be that plants required more transcriptional regulatory genes expression in response to pathogen invasion and spread during the early and mid-stages, while plants already began to resist the pathogens attack or enter the stage of host death in the late-stage infection, leading to reduced gene expression. TFs are key regulators of plant responses to environmental signals, directly influencing target genes by binding to specific cis-regulatory elements in their promoters (Riechmann, 2018). Therefore, we further performed TFs analysis of all DEGs. The results showed that a total of 52, 59 and 56 TF families annotated by DEGs identified in LYBH2 vs. LYBH1, LYBH3 vs. LYBH1 and LYBH4 vs. LYBH1, respectively. The most common TFs detected included AP2/ERF-ERF, bHLH, NAC, MYB, Tify, and WRKY. The WRKY family is one of the largest families of specific TFs that regulate various defense processes and play crucial roles in regulating defense-related genes by binding to W-Box cis-elements (Rushton et al., 1996; Rushton et al., 2010). Certain WRKY members have been shown to be important in lily plants defense against *F. oxysporum*. For instance, Deng et al. reported that the *LrWRKY11* positively regulated Lilium regale resistance to *F. oxysporum* by interacting with salicylic acid/jasmonic acid signaling pathways and modulating *LrDIR1* expression to increase lignin/lignans accumulation (Deng et al., 2023). Additionally, Karre. et al. identified that silencing the *HvWRKY23* gene increased the proportion of diseased spikelets and fungal biomass (Karre et al., 2019). Our study identified bHLH and AP2/ERF-ERF as first two families with more TFs, with significant upregulation in all three comparisons. bHLH, the second largest TF family, is known for its role in transcription and protein-interacting regulation in plants (Jin et al., 2021). Similarly, AP2/ERF TFs are crucial in regulating various disease resistance pathways (Licausi et al., 2013). The AP2/ERF family can be categorized into ERF, DREB, AP2, and RAV subfamilies based on conserved DNA binding domains, with ERF specifically involved in plant defence responses to pathogens via SA and ethylene (ET)/JA-dependent signal transduction pathways (Ma R. et al., 2017). In *F. oxysporum*-inoculated lilies, genes encoding WRKY and ERF families showed increased expression levels (He et al., 2019). Consequently, it was suggested that the differential regulation of these TFs may trigger the production of antimicrobial compounds as a defence mechanism against pathogens.

The changes of this transcriptome were related to the metabolome during the infection and spread of pathogenic bacteria. To prevent the invasion of pathogens, plants will mobilize a large number of defense factors, especially the second metabolites, such as reactive oxygen species and phytoalexins (Kumudini et al., 2018). Phytoalexins are primarily synthesized through the phenylpropanoid metabolism pathway, including flavonoids, terpenoids, alkaloids, and phenolic acids (Ahuja et al., 2012). Metabolomics results indicated that the relative content of two phenolic acids (p-Coumaroylmalic acid and 1-O-p-Coumaroyl-β-D-glucose), N-(malonyl) phenylalanine and juglanoside D continuously increased with the severity of the disease. In addition, some metabolites were identified only in one of the stages of infection, such as the kaempferol-3-O-rutinoside-7-O-rhamnoside was especially discovered in LYBH2. Ma et al. found that the kaempferol 3-O-rutinoside, a flavonoid and derivative of quercetin, had certain anti-bacterial effect (Ma Y. et al., 2017). The quercetin-3-O-glucoside (isoquercitrin) was only in LYBH3 and downregulated, which was consistent with the results of previous studies (Wei et al., 2021). The quercetin-3-O-galactoside (hyperin) and apigenin-5-O-glucoside were unique to LYBH4. Previous studies have shown that flavonoids including quercetin, apigenin, rutin and naringenin are known for their antiviral properties, their mechanism of action involves inhibiting viral neuraminidase, proteases, and DNA/RNA polymerases that interact with viral proteins (Ninfali et al., 2020). Additionally, they can enhance plant tissue responses to pathogenic microorganisms, thereby improving disease resistance (Gatto et al., 2011). Therefore, the changes in the relative content of these metabolites may lead to enhance plant disease resistance.

The combined transcriptomic and metabolomic analyses revealed that phenylpropanoid biosynthesis was significantly enriched in both the transcriptomic and metabolomic data, indicating this pathway was crucial in Longya lily response to bulb rot. In response to pathogen infection, there were 55 DEGs involved in this pathway, and found these DEGs were involved in the synthesis of various important proteins, such as *PAL*, *CSE*, *CCoAOMT*, *COMT* and *POD*, which were upregulated in LYBH2, LYBH3 and LYBH4. However, some DEGs encoding the cinnamate 4-Hydroxylase (*CYP73A*), 4-coumarate-CoA ligase (*4CL*), shikimate O-hydroxycinnamoyl transferase (*HCT*), cinnamoyl-CoA reductase (*CCR*) and cinnamyl-alcohol dehydrogenase (*CAD*) were downregulated in LYBH2, LYBH3 and LYBH4. Phenylpropanoid metabolism is a critical secondary pathway in plants, giving rise to flavonoids, lignans, cinnamic acids, amides, and other metabolites. This pathway initiates with key enzymes *PAL*, *CYP73A* and *4CL*, leading to the production of cinnamic acid and p-coumaroyl-CoA, which further branches into the phenylalanine metabolic pathway and the flavonoid metabolic pathway (Dixon et al., 2002). The *PAL*, *CYP73A* and *4CL* enzymes play a significant role in the synthesis of phenolic compounds involved in plant defense mechanisms. And the activities of these enzymes aligned closely with the expression of their respective genes, potentially leading to consistent accumulation of the catalytic products. The *POD* is involved in lignin synthesis, which provides a structural barrier for the plant against the pathogen (Sun et al., 2024). And the increase of its activity can promote the oxidation of phenol to quinone, which is harmful to pathogenic bacteria (Lu et al., 2020). Phenylpropanoid metabolites play a key role in both plant development and interactions with the environment (Yadav et al., 2020). In this study, we discovered five upregulated metabolites associated with bulb rot in Longya lily, including L-tyrosine, trans-5-O-(p-Coumaroyl) shikimate, sinapaldehyde, 1-O-sinapoyl-β-D-glucose and coniferin. L-tyrosine is the precursor of lignin, which can prevent the penetration of bacteria through strengthening the cell wall, affecting the formation of the haustorium within the cell (Kutschera et al., 2019). Coniferyl alcohol is converted to coniferin by *UGT72E*. Coniferyl alcohol is one of the major monomers of lignin, and coniferin is a plausible monolignol precursor in the lignification of plant cell walls (Guan et al., 2022), and one study was found to inhibit fungal growth and melanization (Kumar et al., 2020). Interestingly, despite significant changes in gene expression profiles, there were no notable differences in metabolite levels between healthy and infected bulbs, suggesting that environmental factors, structural genes, and TFs may play a crucial role in the biosynthesis and accumulation of metabolites.

## 5 Conclusion

We studied the variations of gene expression and metabolite in Longya lily bulbs with different degrees of disease using transcriptome and metabolomics analyses. The results indicated differential expression of genes during disease progression, influencing the composition and content of certain DAMs like quercetin, apigenin, kaempferol, and coniferin. The combined KEGG enrichment results indicated that phenylpropanoid biosynthesis pathways may play essential roles in fighting against bulb rot disease. This study lays the foundation for understanding the molecular mechanisms of Longya lily’s resistance to bulb rot disease and offers insights for breeding resistant lily varieties. The potential roles of the identified key regulatory genes and metabolites in disease resistance outlined in this study may need further exploration and validation.

## Data Availability

The datasets presented in the study are publicly available. This data can be found here: NCBI SRA repository under the project number PRJNA1143388 (https://www.ncbi.nlm.nih.gov/sra/PRJNA1143388).
